# Multiscale Region-Level VHR Image Change Detection via Sparse Change Descriptor and Robust Discriminative Dictionary Learning

**DOI:** 10.1155/2015/947695

**Published:** 2015-03-30

**Authors:** Yuan Xu, Kun Ding, Chunlei Huo, Zisha Zhong, Haichang Li, Chunhong Pan

**Affiliations:** National Laboratory of Pattern Recognition, Institute of Automation, Chinese Academy of Sciences, Beijing 100190, China

## Abstract

Very high resolution (VHR) image change detection is challenging due to the low discriminative ability of change feature and the difficulty of change decision in utilizing the multilevel contextual information. Most change feature extraction techniques put emphasis on the change degree description (i.e., in what degree the changes have happened), while they ignore the change pattern description (i.e., how the changes changed), which is of equal importance in characterizing the change signatures. Moreover, the simultaneous consideration of the classification robust to the registration noise and the multiscale region-consistent fusion is often neglected in change decision. To overcome such drawbacks, in this paper, a novel VHR image change detection method is proposed based on sparse change descriptor and robust discriminative dictionary learning. Sparse change descriptor combines the change degree component and the change pattern component, which are encoded by the sparse representation error and the morphological profile feature, respectively. Robust change decision is conducted by multiscale region-consistent fusion, which is implemented by the superpixel-level cosparse representation with robust discriminative dictionary and the conditional random field model. Experimental results confirm the effectiveness of the proposed change detection technique.

## 1. Introduction

Remote sensing image change detection (CD) aims to identify the land cover changes from the coregistered multitemporal images. It has extensive applications such as damage assessment [1] and forest monitoring [2]. Over the past decades, great efforts have been made to detect changes from the images of different resolutions [2–5]: low, moderate, high, and even very high resolution. Change detection, especially for very high resolution (VHR) images, is an ongoing hot topic in remote sensing image processing [5–7].

In the remote sensing literature, traditional CD approaches [4–6, 8–10] usually consist of two sequential steps: change feature extraction and change decision. The change features are often organized based on visual features, like spectral feature, Gabor feature, and morphological feature. Such features try to describe the local structure of images. Among them, the filter-based features especially Gabor wavelet and morphological profiles have been proved to perform well on the task of change detection and hyperspectral classification [7, 11, 12]. The underlying reason is that the local structure can be well captured by different frequency components and the false alarms are significantly reduced by taking the spatial-contextual information into consideration. Furthermore, the morphological profile (MP) feature [12] draws much attention due to the nonlinear nature of morphological operations.

Having the visual features extracted, many approaches explicitly define the change features to describe the change signatures at a pixel or within a small region [8–10, 13, 14]. The most common strategy to design the change feature is differentiating [8, 9, 15] or stacking [6] the pairwise bitemporal visual features. Change feature built upon the differentiating operation directly reflects the change degree; that is, the difference feature vectors from the changed region usually present larger magnitudes than those from the unchanged region. However, the change feature by differentiating lacks the robustness to registration noise and viewpoint variation. In contrast, the change feature derived by stacking is more robust to the above impacts at the cost of higher feature dimension.

Subsequently, change decision is required to generate the change map (CM). In general, existing approaches can be categorized into two groups.

*(1) Thresholding.* This group of methods are based on thresholding the difference image. Image differencing [16] and change vector analysis [8] are the representatives. They are simple and easy to interpret results; however, low precision especially on high resolution images is their drawback.

*(2) Clustering or Classification.* This group of algorithms are mainly based on the clustering or classification techniques. For example, Celik [15] applied *K*-means to cluster the spectral difference features, and promising performances were achieved on multispectral images. Recently, Volpi et al. [17] extended this method to the nonlinear case. However, these unsupervised methods are less effective for high resolution images that contain complex changes. Meanwhile, the performances by the unsupervised methods can vary with different change features and the results sometimes lack meaningful interpretation.

To tackle these issues, the supervised and semisupervised classifiers are introduced for change detection. For example, support vector machine (SVM) was utilized to classify change features in [6] and a transition matrix indicating the “from-to” changes was derived. As the scarcity of training sample may lead to low generalization performance, Bovolo et al. [18] employed the transductive SVM, a semisupervised learner, to classify change features. By exploiting the user-labeled samples, the supervised CD methods are superior to the unsupervised ones in diminishing the semantic gap between the change features and the real changes and in explaining the change maps semantically.

Despite the novelties of the traditional change features and the change decision strategies in the literature, they are inadequate for VHR image change detection due to the following two factors.

(1) In general, change feature contains the following two components: change degree description (CDD, i.e., in what degree the changes have happened) and change pattern description (CPD, i.e., how the changes changed). The two components are equally important for characterizing changes. However, the traditional change features treat them overwhelmingly unequally; that is, the latter component is usually being ignored.

(2) Both the multiscale and the region-consistency characteristics are important for the reliable change detection. However, the traditional change decision procedures seldom consider them simultaneously.

In this paper, the above two items are considered carefully. The rationale of the proposed approach includes the following.

(1) Change degree description and change pattern description are integrated seamlessly. Intuitively, if two image regions are similar, the features from one region can sparsely represent the features from the other with the low errors and vise versa. Compared to the simple Euclidean distance, the sparse representation error is more robust to the illumination change, seasonal variation, and registration noise. Therefore, it is a suitable candidate for describing the change degree. On the other hand, since MP is good at representing the local appearance of high resolution images, it is computed at each position and stacked with the change degree descriptor to generate the final change feature, sparse change descriptor (SCD). Compared to the traditional change descriptors [6, 8, 9, 13], SCD captures both the change degree and change pattern.

(2) The simultaneous consideration of the region-consistency and the multiscale characteristic of the changes is implemented by the region-level cosparse representation and conditional random field (CRF) fusion. In detail, the proposed change decision strategy starts with constructing a bitemporal image pyramid of the considered images. At each scale of the pyramid, the bitemporal images are cosegmented into small parcels (i.e., superpixels or image objects, or segmentation regions). For each pair of the corresponding parcels from some scale, its probability that belongs to the changed class is predicted by the isotonic regression model [19] trained on the labeled sparse representation errors. These errors are computed by the cosparse representation with the learned robust discriminative dictionaries (RDD), an improved version of the traditional dictionaries [20, 21]. The robustness is realized by weighting the features. Once the change probability maps for all scales are estimated, they are fed into CRF to merge the information from different scales and smooth the resultant change map.

Compared with the related approaches, the contributions of this paper lie in the following aspects.

- A new change feature, SCD, is proposed, which represents both the change degree and change pattern feature.
- A robust discriminative dictionary learning (RDDL) model is presented, which is resistant to outliers in modeling the change feature distribution.
- The cosparse representation is resorted to predict the change probability for all pixels within an image object, which makes the detected changes more consistent within a homogeneous region.
- CRF is used to fuse the change detection results from different scales, which makes the proposed method capable of recognizing the changes from different scales.

The rest of this paper is organized as follows: Section 2 illustrates the proposed CD method, Section 3 discusses the parameter estimation problem, Section 4 presents the experimental results and analysis, and finally Section 5 concludes the paper.

## 2. The Proposed Approach

**Figure 1:**
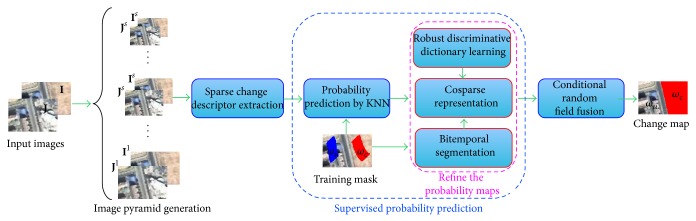
The flowchart of the proposed approach.

As illustrated by Figure 1, the proposed VHR image CD approach consists of the following four steps: pyramid generation, sparse change descriptor extraction, supervised probability prediction, and conditional random field fusion. In the following, we will describe our CD approach in detail.

### 2.1. Pyramid Generation

The objects in an image and the changes between the bitemporal images are highly dependent on the scale, and multiscale analysis is helpful to improve the performance. For this reason, multiscale images are firstly generated by the pyramid decomposition. In detail, the images **I** ^*s*+1^ and **J** ^*s*+1^ at the (*s* + 1)th scale (*s* = 1,…, *S* − 1) of the pyramid are built by downsampling the images **I** ^*s*^ and **J** ^*s*^ at the *s*th scale with the downsampling ratio *ρ* (*ρ* > 1), respectively. Here, **I** ^1^ = **I** and **J** ^1^ = **J** are the original coregistered bitemporal images, which both have the size *H* × *W* × *B*. *H*, *W*, and *B* (*B* = 3 in this paper) are the number of rows, columns, and channels, respectively. Obviously, the sizes of images **I** ^*s*^ and **J** ^*s*^ are *H* ^*s*^ = round(*H*/*ρ* ^*s*−1^) rows, *W* ^*s*^ = round(*W*/*ρ* ^*s*−1^) columns, and *B* channels, where round(·) is the rounding function.

### 2.2. Sparse Change Descriptor Extraction

One of the main difficulties related to the change detection of VHR images lies in the lack of separability of change features caused by the abundant details and the low spectral resolution [5]. In this paper, the discriminative ability of change feature is improved by the sparse change descriptor (SCD). Different from the classical change features [8, 9, 13], two components are contained in SCD: the change degree description (CDD) and the change pattern description (CPD).

#### 2.2.1. Change Degree Description

Motivated by the state-of-the-art performance of the sparse-representation-based classifier [22] in face recognition, where the images have serious occlusion and lighting change, the sparse representation error is used as the change degree description. In other words, the magnitude of this error is able to indicate the degree that a test face belongs to a person.

The sparse representation errors are computed on all scales of the image pyramid. At each position of the pairwise images from some scale, two informative representation errors are derived based on two local dictionaries. For this purpose, 3D patches are collected as follows.

1. At the pixel (*i*, *j*) of the image **J** ^*s*^, a 3D patch of size *m* × *m* × *B* (*m* is an odd) is picked and converted to a *m* ^2^ *B* × 1 vector, **y**_*ij*_^*s*^. At the index range {(*i*′, *j*′)∣*i* − *m* ≤ *i*′ ≤ *i* + *m*, *j* − *m* ≤ *j*′ ≤ *j* + *m*} of image **J** ^*s*^, all the 3D patches of size *m* × *m* × *B* are collected and converted to vectors.
2. These vectors are arranged by column to build the local dictionary **D**_*J*,*ij*_^*s*^ of the image **J** ^*s*^ at (*i*, *j*). Obviously, the size of this dictionary is *m* ^2^ *B* × (*m* + 2)^2^.
3. Similarly, **x**_*ij*_^*s*^ and **D**_*I*,*ij*_^*s*^ can also be constructed from the image **I** ^*s*^.

Based on the dictionaries **D**_*I*,*ij*_^*s*^ and **D**_*J*,*ij*_^*s*^, two representation errors are computed: the error *e*_*I*,*ij*_^*s*^ of **x**_*ij*_^*s*^ under **D**_*J*,*ij*_^*s*^ and the error *e*_*J*,*ij*_^*s*^ of **y**_*ij*_^*s*^ under **D**_*I*,*ij*_^*s*^. To compute these errors, the features **x**_*ij*_^*s*^ and **y**_*ij*_^*s*^ are expanded under the dictionaries **D**_*J*,*ij*_^*s*^ and **D**_*I*,*ij*_^*s*^, respectively. Specifically,

(1) α^ijs=argminα12xijs−DJ,ijsα22+λα1,β^ijs=argminβ12  yijs−DI,ijsβ22+λβ1,

where ‖**x**‖_*p*_ = (∑_*i*_|*x*_*i*_|^*p*^)^1/*p*^ (*p* > 0) denotes the *ℓ*_*p*_-norm of vector **x**, ${\hat{\alpha }}_{ij}^{s}$ and ${\hat{\beta }}_{ij}^{s}$ are the optimal representation vectors, and *λ* controls the sparsity of representation coefficients.

Based on ${\hat{\alpha }}_{ij}^{s}$ and ${\hat{\beta }}_{ij}^{s}$, *e*_*I*,*ij*_^*s*^ and *e*_*J*,*ij*_^*s*^ can be computed by

(2) eI,ijs=xijs−DJ,ijs·α^ijs22,eJ,ijs=yijs−DI,ijs·β^ijs22.

To build the change degree feature for the *s*th scale, we stack the two errors; that is, **e**_*ij*_^*s*^ = [*e*_*I*,*ij*_^*s*^, *e*_*J*,*ij*_^*s*^]^*T*^ (*T* denotes the transposition). Obviously, larger values of these errors indicate more remarkable changes and vise versa.

#### 2.2.2. Change Pattern Description

As mentioned before, the morphological profile feature [12] has been proved adept at describing the local structure of images; therefore, it is adopted in our change pattern description. For an image **I** with *B* channels, let us denote the morphological profile feature at pixel (*i*, *j*) of scale *s* as **m** **p**_*I*,*ij*_^*s*^ = [(**m** **p**_*I*,*ij*_^*s*,1^)^*T*^,…, (**m** **p**_*I*,*ij*_^*s*,*b*^)^*T*^,…, (**m** **p**_*I*,*ij*_^*s*,*B*^)^*T*^]^*T*^, where **m** **p**_*I*,*ij*_^*s*,*b*^ is the morphological profile feature of image **I** at the *s*th scale and the *b*th channel.

Based on the change degree description and the change pattern description, the SCD of the bitemporal images **I** and **J** at pixel (*i*, *j*) and scale *s* is the concatenation of the feature **e**_*ij*_^*s*^ with **m** **p**_*I*,*ij*_^*s*^ and **m** **p**_*J*,*ij*_^*s*^; that is,

(3) $$\begin{array}{c} f_{ij}^{s}={\left[{\left(e_{ij}^{s}\right)}^{T},{\left(mp_{I,ij}^{s}\right)}^{T},{\left(mp_{J,ij}^{s}\right)}^{T}\right]}^{T}. \end{array}$$

At each scale, two structuring elements (SEs) with the radius parameters *r* = 0, (*m* − 1)/2 are used. Here, *r* = 0 denotes the original spectral features.

By combining the change degree descriptor and the change pattern descriptor, a powerful change feature, SCD, is formed. The change degree component makes it robust to false changes and the change pattern component improves its descriptive power. By this change feature, the change decision in the following sections can be made more reliably.

### 2.3. Supervised Probability Prediction

This step is aimed at estimating a probability map that indicates the change state of all pixels in each scale of the image pyramid. These maps will be used in the CRF model to generate the final change map. For estimating these probability maps reliably, we employ the cosparse representation model [23] which can exploit the spatial-contextual information effectively. Before that, two conditions should be satisfied.

*Condition 1*:* Training Robust and Discriminative Dictionaries.* Good dictionaries would produce the representation errors that have strong discrimination ability and stability, which are both helpful for reliable probability prediction. One simple strategy for dictionary learning is to train a dictionary on the given training data for each class separately. However, this strategy has at least three drawbacks: (1) the scarcity of the labeled data cannot ensure the generalization ability of the learned dictionaries; (2) the relationship between the dictionaries of different classes is ignored; (3) a few mislabeled data may mislead the dictionary learning process. To deal with these problems, an “add-and-remove” strategy for dictionary training is proposed. It consists of two basic steps: (1) enlarge the amount of samples on the basis of the user-labeled data by a preclassification process (“add” step, see Section 2.3.1) and (2) train all dictionaries simultaneously on the augmented and weighted training data. The weights are updated gradually to remove the intraclass outliers (“remove” step, see Section 2.3.2).

*Condition 2*:* Estimating the Probability that Pairwise Pixels Belong to the Changed Class Based on the Representation Errors Obtained by the Cosparse Representation.* One can either use a parametric (e.g., logistic regression) or a nonparametric (e.g., isotonic regression [19]) model to predict this probability. Considering the nonparametric model is distribution-free and has the advantage in reducing the number of parameters to be adjusted (e.g., regularization parameter in parametric model), it is used in the probability prediction step (see Sections 2.3.2 and 2.3.3).

In the following three parts, the proposed supervised probability prediction method is elaborated step by step.

#### 2.3.1. Initial Probability Prediction by KNN

This is the “add” step, which is aimed at preclassifying the extracted change descriptors based on the user-labeled data and making a new training set for all scales. Furthermore, each training sample is allocated with an initial weight, which will be used in the proposed robust discriminative dictionary learning model.

For the change feature **f**_*ij*_^*s*^ extracted at the position (*i*, *j*) from the images **I** ^*s*^ and **J** ^*s*^, *k* nearest neighbors are found from the provided training set of the scale *s*. Assume that there are *k*_*c*_ neighbors of the changed class (*ω*_*c*_) and *k*_*u*_ neighbors of the unchanged class (*ω*_*u*_). The probability of **f**_*ij*_^*s*^ belonging to *ω*_*c*_ can be estimated by *p*(*ω*_*c*_∣**f**_*ij*_^*s*^) = *k*_*c*_/*k*. After computing the probability for each position, a coarse probability map **c** **P** **M** ^*s*^ is obtained.

**Figure 2:**
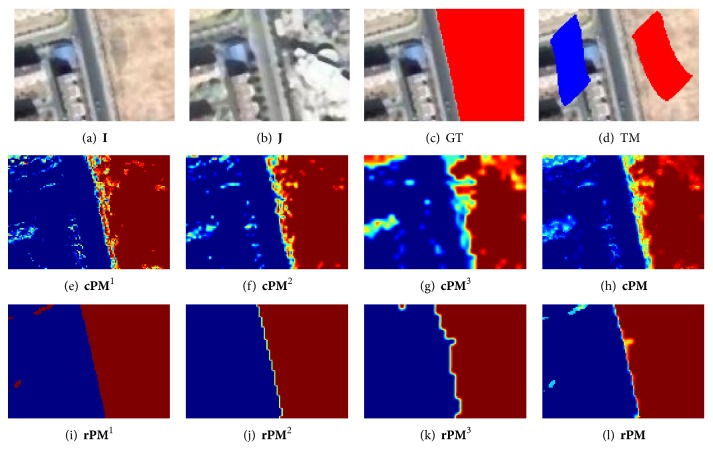
A toy example for estimating the probability maps. (a)–(d) Image **I**, image **J**, ground truth (GT) image that is superposed on **I** with red, and training mask (TM) image with red and blue denote the labeled changed and unchanged pixels, respectively. (e)–(g) The estimated coarse probability maps **c** **P** **M** ^*s*^ (*s* = 1,2, 3) by KNN and (h) the weighted sum of them, **c** **P** **M**. (i)–(k) The refined probability maps **r** **P** **M** ^*s*^ (*s* = 1,2, 3) and (l) the weighted sum of them, **r** **P** **M**.

To incorporate the relationship between different scales, all the probability maps are resized to *H* × *W* and combined into **c** **P** **M** = ∑_*s*=1_^*S*^ *κ* ^*s*^UP(**c** **P** **M** ^*s*^, *ρ* ^*s*−1^), where *κ* ^*s*^ is the weight for the *s*th scale; UP(**I**, *ρ*) is the operation that upsamples the image **I** with the upsampling ratio *ρ*. It is reasonable to set the maps that have higher confidence (reflected by the total classification accuracy) with higher weights. The adaptive weights are computed as follows:

(4) $$\begin{array}{c} \kappa^{s}=\frac{1-\text{TF}{\text{R}}^{s}}{S-\sum_{s=1}^{s}\text{TF}{\text{R}}^{s}},\;s=1,\ldots ,S, \end{array}$$

where TFR^*s*^ is the total false rate of the probability map at the scale *s*, which is achieved by comparing the ground truth labels of the training samples and the predicted labels by KNN. After that, **c** **P** **M** is downsampled to different scales to replace the original **c** **P** **M** ^*s*^ (*s* = 1,…, *S*).  Figure 2 gives the estimated probability maps for a toy data set. Figures 2(e)–2(h) show the probability maps before using multiscale fusion and the weighted sum of them. From these figures, the weighted sum is more accurate than each monoscale estimation.

#### 2.3.2. Robust Discriminative Dictionary Learning

After computing the coarse probability maps, the dictionaries can be learned at each scale based on the augmented and weighted training sets (the “remove” step). The new training sets are defined as *𝒳*_*c*_^*s*^ = {(*i*, *j*)∣cPM_*ij*_^*s*^ > 0.5} and *𝒳*_*u*_^*s*^ = {(*i*, *j*)∣cPM_*ij*_^*s*^ ≤ 0.5} for the changed class and unchanged class, respectively. The weight for a sample in *𝒳*_*c*_^*s*^ is *w*_*ij*_^*s*^ = cPM_*ij*_^*s*^ and that for a sample in *𝒳*_*u*_^*s*^ is *w*_*ij*_^*s*^ = 1 − cPM_*ij*_^*s*^.

For convenience, the scale symbol *s* is omitted in the following sections except for specific explanation. *𝒳*_*c*_ and *𝒳*_*u*_ may contain some outliers, which would undermine the discriminative ability of the learned dictionaries **D**_*c*_ and **D**_*u*_ (for the classes *ω*_*c*_ and *ω*_*u*_, resp.). To solve this problem, each sample is weighted and the weight is updated during dictionary training. By this way, intraclass outliers can be stably removed and the discriminative ability of dictionaries is simultaneously kept.

For this purpose, we build a nonparametric model between *w*_*ij*_ and the corresponding representation errors. Here, the isotonic regression model [19, 24] is adopted as it can fit an isotonic function without any assumption about the specific form. For (*i*, *j*) ∈ *𝒳*_*c*_, the mapping is *w*_*ij*_ = IR(SRF(*e*_*ij*_^*c*^, *e*_*ij*_^*u*^)); for (*i*, *j*) ∈ *𝒳*_*u*_, the mapping is *w*_*ij*_ = 1 − IR(SRF(*e*_*ij*_^*c*^, *e*_*ij*_^*u*^)). The class-specified representation errors *e*_*ij*_^*c*^ and *e*_*ij*_^*u*^ are defined as

(5) $$\begin{array}{c} e_{ij}^{c}={\left\|f_{ij}-D_{c}{\hat{\alpha }}_{ij}^{c}\right\|}_{2}^{2},\;\;e_{ij}^{u}={\left\|f_{ij}-D_{u}{\hat{\alpha }}_{ij}^{u}\right\|}_{2}^{2}, \end{array}$$

where ${\hat{\alpha }}_{ij}^{c}$ and ${\hat{\alpha }}_{ij}^{u}$ are the best representation vectors of **f**_*ij*_ under **D**_*c*_ and **D**_*u*_, respectively. IR(·) is an isotonic regression function with single variable [19, 24]. SRF(·, ·) is named the signed ratio function, which is defined as

(6) $$\begin{array}{c} \text{SRF}\left(x,y\right)=\mathrm{sgn}\left(x-y\right)\left(\frac{\max\left(x,y\right)}{\min\left(x,y\right)}-1\right),\;x,y>0, \end{array}$$

where sgn(*x* − *y*), max⁡(*x*, *y*), and min⁡(*x*, *y*) denote the sign of *x* − *y* and the maximal and minimal value between *x* and *y*, respectively. The role of the SRF function is to convert the complex bivariate case to the tractable univariate case by considering the relationship between *e*_*ij*_^*c*^ and *e*_*ij*_^*u*^; that is, if one of them is large the other would be small, and vise versa.

Given a set of labeled triplets, {(*e*_*ij*_^*c*^, *e*_*ij*_^*u*^, label_*ij*_)} (label_*ij*_ is 1 if the pixel (*i*, *j*) is in the user-specified changed regions and 0 if it is in the user-specified unchanged regions), we first compute the SRF values, SRF(*e*_*ij*_^*c*^, *e*_*ij*_^*u*^). The generalized pool-adjacent-violators algorithm (GPAV) [24] is subsequently adopted to fit the univariate isotonic function IR(·) on {(SRF(*e*_*ij*_^*c*^, *e*_*ij*_^*u*^), label_*ij*_)}. Using the fitted function (i.e., a staircase function), the label value can be evaluated on all pixels. They are used to build the weights of the reconstruction terms in the following robust discriminative dictionary learning (RDDL) model:

(7) min⁡Dc,Du∈Ω,Ac,Au,{wij} 1Nc∑(i,j)∈Xc12wijfij−Dcαij22+λ1αij1min⁡Dc,Du∈Ω,Ac,Au,{wij} +1Nu∑(i,j)∈Xu12wijfij−Duαij22+λ1αij1min⁡Dc,Du∈Ω,Ac,Au,{wij} +λ2tr⁡(DcTDu)s.t.   wij=IRSRFeijc,eiju, ∀i,j∈Xc,s.t.   wij=1−IR(SRF(eijc,eiju)), ∀(i,j)∈Xu,

where *Ω* is a convex set that consists of matrices and each column of each matrix has the *ℓ*_2_-norm less than 1. *N*_*c*_ and *N*_*u*_ are the number of samples in *𝒳*_*c*_ and *𝒳*_*u*_, respectively, **A**_*c*_ and **A**_*u*_ collect all representation coefficients from *ω*_*c*_ and *ω*_*u*_, respectively, *λ*_1_ and *λ*_2_ are the trade-off parameters, and tr⁡(·) is the trace operator.

We propose using the alternative coordinate descent technique [20, 21] to solve the above problem. It consists of the following three steps.

*Step 1.* Update **A**_*c*_ and **A**_*u*_ with the fixed **D**_*c*_, **D**_*u*_, {*w*_*ij*_}. We update the columns of **A**_*c*_ and **A**_*u*_ separately. Actually, only the following problem needs to be solved:

(8) $$\begin{array}{c} \underset{\alpha_{ij}}{\min}\frac{1}{2}w_{ij}{\left\|f_{ij}-D\alpha_{ij}\right\|}_{2}^{2}+\lambda_{1}{\left\|\alpha_{ij}\right\|}_{1}, \end{array}$$

where **D** = **D**_*c*_ if (*i*, *j*) ∈ *𝒳*_*c*_ and **D** = **D**_*u*_ otherwise. The least angle regression algorithm [25] is adopted to solve this subproblem due to its computational efficiency. Once all *α*_*ij*_ are obtained, we can get the new **A**_*c*_ and **A**_*u*_.

*Step 2.* Update **D**_*c*_, **D**_*u*_ with the fixed **A**_*c*_, **A**_*u*_, {*w*_*ij*_}. The involved subproblem is

(9) min⁡Dc,Du∈Ω1Nc∑(i,j)∈Xc12wijfij−Dcαij22+λ2tr⁡(DcTDu)hhhhh+1Nu∑(i,j)∈Xu12wijfij−Duαij22.

**D**_*c*_ and **D**_*u*_ can be separately updated by the projection gradient descending procedure [26].

*Step 3.* Update {*w*_*ij*_} with the fixed **D**_*c*_, **D**_*u*_, **A**_*c*_, and **A**_*u*_. With the updated representation coefficients **A**_*c*_ and **A**_*u*_, the representation errors *e*_*ij*_^*c*^ and *e*_*ij*_^*u*^ can be computed for each pixel by (5). Given the labeled triplets {(*e*_*ij*_^*c*^, *e*_*ij*_^*u*^, label_*ij*_)}, a new isotonic regression model IR(SRF(*e*_*ij*_^*c*^, *e*_*ij*_^*u*^)) can be fitted. Based on this model, the weights are updated by

(10) wij⟵IR(SRF(eijc,eiju)), ∀(i,j)∈Xc,wij⟵1−IR(SRF(eijc,eiju)), ∀(i,j)∈Xu.

The above steps are iterated alternatively until the solution keeps stable.

By the robust discriminative dictionary learning, two dictionaries, **D**_*c*_ and **D**_*u*_, are learned for each scale. And these dictionaries will be utilized in the following superpixel-level cosparse representation step.

#### 2.3.3. Superpixel-Level Cosparse Representation

The role of the cosparse representation is to use the learned dictionaries in the former step to estimate a more accurate probability map for each scale. With the help of these maps, a better data term for the subsequent CRF model can be built, which enables us to further enhance the change detection performances. Since the traditional sparse representation models [22, 27] treat samples independently, they will generate noisy CMs. Thus, we run the cosparse representation [23] on each segmentation region to encode all the features within it simultaneously.

Before cosparse representation, the images **I** and **J** (of some scale *s*) are cosegmented to generate small homogeneous regions. We first segment each image individually by the simple linear iterative clustering (SLIC) algorithm [28] and then obtain the final regions by merging the two segmentation results using the same strategy as [5].

Suppose that **I** and **J** are cosegmented into *L* homogeneous regions *ℛ* = {*R*_*l*_∣*l* = 1,…, *L*}, where *R*_*l*_ is the set that collects all indices of the pixels in the *l*th region. If the feature matrix of the region *R* ∈ *ℛ* is **F**, its encoding matrix $\hat{A}$ can be computed by solving

(11) $$\begin{array}{c} \hat{A}=\underset{A}{\text{argmin}}\frac{1}{2}{\left\|F-DA\right\|}_{F}^{2}+\frac{\lambda_{1}}{\sqrt{\left|R\right|}}{\left\|A\right\|}_{\text{2,1}}\text{,} \end{array}$$

where *λ*_1_ is the regularization parameter that controls the sparsity of **A**, |*R*| denotes the number of features in region *R*, ‖·‖_*F*_ is the Frobenius norm, ‖**A**‖_2,1_ is the *ℓ*_2,1_-norm of **A**, which sums the *ℓ*_2_-norm of all its rows, and **D** is defined as **D** = [**D**_*c*_, **D**_*u*_, **E**], where **E** is an identity matrix.

Let us denote the *j*th column of **F** by **f**_*j*_ and the corresponding representation coefficient vector by ${\hat{\alpha }}_{j}={\left[{\left({\hat{\alpha }}_{j}^{c}\right)}^{T},{\left({\hat{\alpha }}_{j}^{u}\right)}^{T},{\left({\hat{\alpha }}_{j}^{e}\right)}^{T}\right]}^{T}$, where ${\hat{\alpha }}_{j}^{c}$, ${\hat{\alpha }}_{j}^{u}$, and ${\hat{\alpha }}_{j}^{e}$ are the coefficient vectors corresponding to the submatrices **D**_*c*_, **D**_*u*_, and **E**, respectively. If we define the representation errors $e_{j}^{c}={\left\|f_{j}-D_{c}{\hat{\alpha }}_{j}^{c}\right\|}_{2}^{2}$ and $e_{j}^{u}={\left\|f_{j}-D_{u}{\hat{\alpha }}_{j}^{u}\right\|}_{2}^{2}$, then the isotonic regression can be used to estimate the probability that a feature **f**_*j*_ belongs to the class *ω*_*c*_; that is, *p*(*ω*_*c*_∣**f**_*j*_) = IR(SRF(*e*_*j*_^*c*^, *e*_*j*_^*u*^)). By computing the probability for each pixel of the image at the scale *s*, a refined probability map **r** **P** **M** ^*s*^ can be obtained. Similar to the technique proposed in Section 2.3.1, a merged probability map **r** **P** **M** can also be computed. Figures 2(i)–2(l) show the refined probability maps by cosparse representation. By comparing the second and the third rows of Figure 2, it can be concluded that the refined probability maps are more accurate than the coarse ones.

### 2.4. Conditional Random Field Fusion

To reduce the salt-and-pepper-like noise contained in the final CM and to merge the information from different sources, the CRF model [29] is resorted. One simple strategy is to build the data term of CRF only by the refined probability map **r** **P** **M**. However, a better choice is to utilize both **r** **P** **M** and **c** **P** **M**; the reason is that they are usually complementary.

Given **c** **P** **M** and **r** **P** **M**, the energy function of CRF is defined as

(12) EC=∑ij[vcF(Cij,cPM)+vrF(Cij,rPM)] +η∑ij,i′j′S(Cij,Ci′j′,cPM,rPM),

where **C** represents the 0-1 label configuration matrix, *η* is the smoothness parameter, and *v*_*c*_ and *v*_*r*_ = 1 − *v*_*c*_ are the weights that balance the effects of the coarse and refined probability maps. *v*_*r*_ should be set larger than *v*_*c*_ because the refined map is usually more reliable in terms of total accuracy. By the simple try-and-error strategy, we found that *v*_*r*_ = 0.8 and *v*_*c*_ = 0.2 are good choices for the used data sets. *F*(·, ·) is the feature function, which is defined as *F*(*C*_*ij*_, **P** **M**) = PM_*ij*_ if *C*_*ij*_ = 0 and *F*(*C*_*ij*_, **P** **M**) = 1 − PM_*ij*_ otherwise. The smoothness term is defined as

(13) $$\begin{array}{c} S\left(C_{ij},C_{i^{\prime }j^{\prime }},cPM,rPM\right)=\delta_{ij,i^{\prime }j^{\prime }}\exp\left(-\gamma d_{ij,i^{\prime }j^{\prime }}\right), \end{array}$$

where *δ*_*ij*,*i*′*j*′_ = 1 if *C*_*ij*_ ≠ *C*_*i*′*j*′_ and *δ*_*ij*,*i*′*j*′_ = 0 otherwise, *γ* is related to the kernel width, and *d*_*ij*,*i*′*j*′_ is the squared Euclidean distance between the spectral features at (*i*, *j*) and (*i*′, *j*′).

Note that the problem in (12) can be solved efficiently by the max-flow algorithm [30] even for the large-size images. The fast speed enables us to tune *η* conveniently.

## 3. Parameter Estimation

There are some important parameters in the proposed CD approach: *λ* in (1), *λ*_1_ in (7) and (11), *λ*_2_ in (7), and the parameters in SLIC. Considering the computational efficiency, *λ*_1_ and *λ*_2_ are set manually, and their sensitivities are analyzed in Section 4.1.1. The remaining parameters are tuned automatically according to some supervised or unsupervised criterions.

### 3.1. Parameter Estimation: *λ*

**Figure 3:**
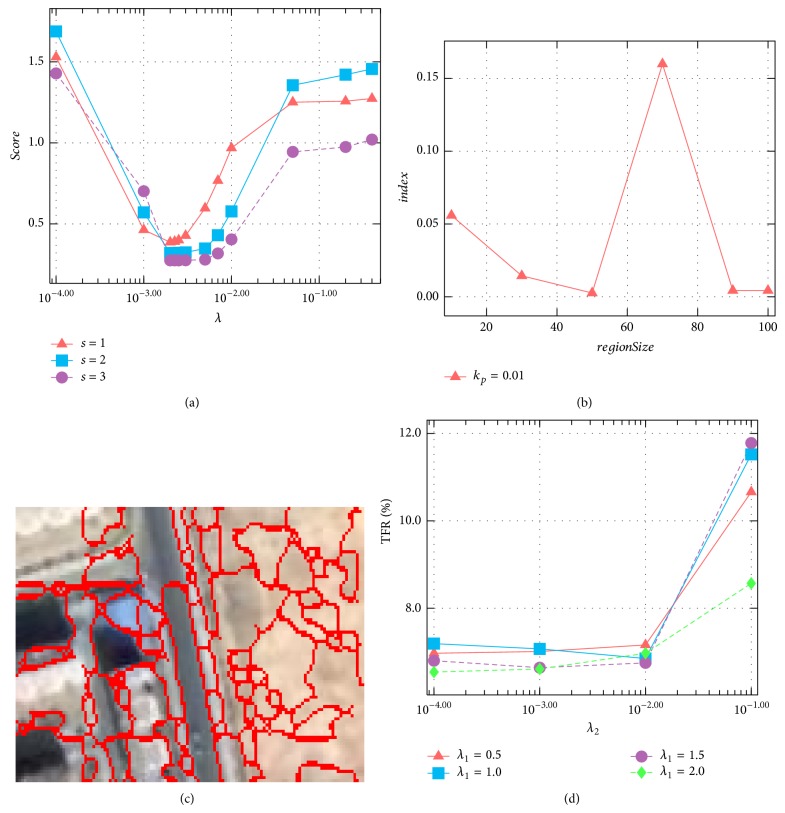
Parameter estimation on toy data set in Figures 2(a)–2(d) (a)–(c) and parameter sensitivity analysis on DS1 (d). (a) The *score*-*λ* curves at three different scales. (b) The *index*-*regionSize* curve at the scale *s* = 1. (c) The segmentation result of the image **I** by the best *regionSize* (i.e., 50 pixels). (d) The relations between *λ*_1_, *λ*_2_ and TFR on DS1.

The selection of *λ* should ensure the change degree feature as discriminative as possible. At each scale *s*, *λ* ^*s*^ for *λ* is estimated separately. Recalling the effectiveness of Fisher discriminant criterion [5, 31] in evaluating the separability of feature, the score function for *λ* ^*s*^ is defined as score (*λ* ^*s*^) = tr⁡(**S**_*w*_(*λ* ^*s*^))/tr⁡(**S**_*b*_(*λ* ^*s*^)), where **S**_*w*_(*λ* ^*s*^) and **S**_*b*_(*λ* ^*s*^) are the intraclass and inter-class scatter matrices of feature *e*_*ij*_^*s*^, respectively. To choose the optimal parameter, *λ* ^*s*^ is discretized at *n* points *λ*_1_^*s*^,…, *λ*_*n*_^*s*^, and the one with the minimal score is chosen as the best parameter. The parameter searching results of the images in Figures 2(a) and 2(b) at three different scales are shown in Figure 3(a). From this figure, the best *λ* for all scales is about 2 × 10^−3^.

### 3.2. Parameter Estimation: *RegionSize* and *k*_*p*_

SLIC [28] has two parameters: *regionSize* and *k*_*p*_. The former determines the minimal area of the segmented regions; the latter is related to the region homogeneity. Smaller *k*_*p*_ generates more homogeneous but more irregular regions. In our change detection task, the regions are expected to be as homogeneous as possible. Therefore, *k*_*p*_ can be fixed as a small value, 0.01, for example, and only *regionSize* needs tuning. Intuitively, large and homogeneous regions are helpful for better performances. Accordingly, the following metric is defined to evaluate the segmentation quality:

(14) $$\begin{array}{c} \text{index}=\frac{1}{L}\overset{L}{\underset{l=1}{\sum }}\frac{1}{\sqrt{n_{l}}}\sqrt{\frac{1}{n_{l}}\underset{t}{\sum }{\left\|x_{lt}-{\bar{x}}_{l}\right\|}_{2}^{2}}, \end{array}$$

where **x**_*lt*_ is the 2*B* dimensional spectral feature that concatenates the spectral features from the two times at the *t*th (*t* ∈ {1,…, *n*_*l*_}) position of the *l*th (*l* ∈ {1,…, *L*}) region, ${\bar{x}}_{l}$ is the average spectral feature of region *l*, and *n*_*l*_ is the area of the *l*th region. The best *regionSize* is the value that minimizes the *index* function. We search this value in the range [10,100].  Figure 3(b) shows the relation between *regionSize* and *index* computed for the images in Figures 2(a) and 2(b) at the first scale. From Figure 3(b), *regionSize* ^1^ = 50 can be regarded as the best parameter for this scale. Running the search algorithm for all scales is time-consuming. Considering the relationship between different scales, we estimate the optimal parameters for the higher scales by *regionSize* ^*s*^ = round(*regionSize* ^1^/*ρ* ^*s*−1^) (*s* = 2,…, *S*).

## 4. Experiments

In this section, four different experiments on three data sets are carried out to test the validity of the proposed techniques.

The first experiment is to evaluate the overall performance of the proposed method. To this end, it is compared with other related CD methods qualitatively and quantitatively (Section 4.1). The second experiment is to validate the effectiveness of multiscale fusion. Hence, the fused results are compared against all monoscale results (Section 4.2). The third experiment is aimed at assessing the effectiveness of the proposed change feature. In particular, SCD is compared with five commonly used change features (Section 4.3). The last experiment makes it possible to validate the effectiveness of the proposed robust discriminative dictionary by comparison with some other dictionaries (Section 4.4).

**Figure 4:**
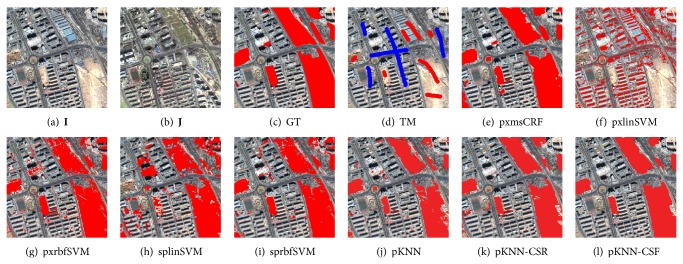
DS1 and the resultant change maps (CMs) by different methods. In the ground truth (GT) images and the CMs, the red regions denote the changed class and the other regions denote the unchanged class. In the training mask (TM) images, the red regions denote the changed class, the blue regions denote the unchanged class, and the remaining regions are unlabeled.

**Figure 5:**
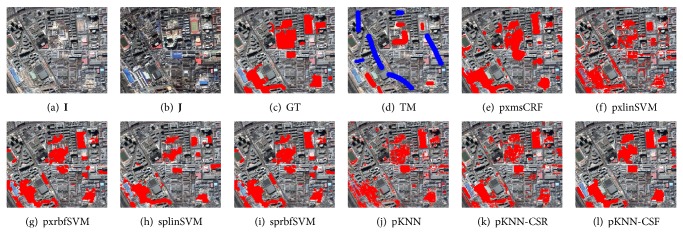
DS2 and and the resultant change maps (CMs) by different methods.

**Figure 6:**
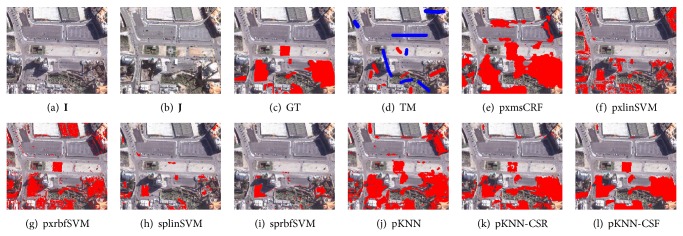
DS3 and and the resultant change maps (CMs) by different methods.

Three data sets named DS1, DS2, and DS3 are used for performance comparison, which were taken by QuickBird 2 satellite over Beijing, China. The sizes of them are 1024 × 1024 pixels, 1001 × 1170 pixels, and 451 × 525 pixels, respectively. As shown in Figures 4(a)–4(d), 5(a)–5(d), and 6(a)–6(d), each data set consists of two coregistered pansharpened images (a) and (b), a reference ground truth (GT) image (c), and a training mask (TM) image (d). The resolution of the pansharpened images is 0.7 m/pixel. In the ground truth images, the changed class is in red. The training mask images are used to generate the training samples of supervised change detection methods. In these images, the changed and unchanged training regions are labeled with red and blue, respectively.

For performance comparison, three metrics are used: false alarm rate (FAR), missed alarm rate (MAR), and total false rate (TFR) [32].

### 4.1. First Experiment: Overall Performance Evaluation

To demonstrate the effectiveness of the proposed CD method, the following methods are used for comparison.

*(i) pxmsCRF.* Similar to our method, we decompose the images into three scales. At each scale, CRF (we use the code provided by http://users.cecs.anu.edu.au/jdomke/JGMT/) is trained to classify the SCD feature extracted at each pixel. The final CM is obtained by the majority voting strategy.

*(ii) pxlinSVM.* It is a multiscale pixel-level CD method. It extracts the MP feature at each pixel using the SE parameters *r* = 0,3, 7,15 and classifies the multiscale features by the linear SVM classifier. The regularization parameter *c* is selected by 5-fold cross-validation.

*(iii) pxrbfSVM.* It is a modified version of pxlinSVM by using the RBF kernel in SVM. Both the regularization parameter *c* and the RBF kernel width *σ* are set via 5-fold cross-validation.

*(iv) splinSVM.* It is an object-level CD method. It first segments the two images **I** and **J** by SLIC. As pxlinSVM, the MP feature is extracted at all pixels. Based on these features, an object-level change feature is computed for each region by the approach used in [5]. The object-level change features are classified by the linear SVM classifier to generate the final CM. There are three tunable parameters in this method, that is, *c* in linear SVM, *regionSize*, and *k*_*p*_ in SLIC. *c* is selected by 5-fold cross-validation; *regionSize* and *k*_*p*_ are searched in the range [10,100]×[10^−3^, 1], and the best test performance is reported.

*(v) sprbfSVM.* In this approach, linear SVM in splinSVM is replaced by the RBF kernel SVM classifier. Both *c* and *σ* are set via cross-validation. Similar to splinSVM, *regionSize* and *k*_*p*_ are also tuned to find the best test performance.

*(vi) pKNN.* **c** **P** **M** is computed by our approach, and the CM is generated by the threshold 0.5 on **c** **P** **M**. Specifically, for the *i*th row and the *j*th column of the change map **C** **M**, CM_*ij*_ = 1 if cPM_*ij*_ > 0.5 and CM_*ij*_ = 0 otherwise.

*(vii) pKNN-CSR.* **r** **P** **M** is computed by our approach, and the CM is generated by the threshold 0.5 on **r** **P** **M**.

*(viii) pKNN-CRF.* pKNN-CRF is the proposed approach. 

In the methods pKNN, pKNN-CSR, and pKNN-CRF, the computation cost of KNN (in Section 2.3.1) increases with the number of training samples. Therefore, instead of using KNN directly, the samples of the changed and unchanged class are clustered into *K*_*c*_ and *K*_*u*_ clusters, respectively. This modified version is called the prototype KNN (pKNN) classifier.

#### 4.1.1. Parameter Settings

As mentioned above, the parameters *λ*, *regionSize*, and *k*_*p*_ in the proposed method can be estimated automatically. In all experiments, the searching ranges for *λ* and *regionSize* are [10^−4^, 0.4] and [10,100], respectively.

The level of the pyramid *S*, the downsampling ratio *ρ*, and the size of image patch *m* should be set according to the image resolution. As all data sets have the same resolution, these parameters are manually set as *S* = 3, *ρ* = 2, and *m* = 7.

The parameters *K*_*c*_ and *K*_*u*_ in pKNN are set as 500. The number of the neighbors *K* for classification in pKNN is set as 7. The regularization parameters *λ*_1_ and *λ*_2_ are set as 1 and 0.01, respectively. In addition, the parameter *η* in CRF is tuned to reach the lowest TFR.

As *λ*_1_ and *λ*_2_ are designated manually, it is necessary to evaluate the influence of them on the final performances. To this aim, the TFRs of pKNN-CSR are computed on the parameter grid {0.5,1, 1.5,2}×{10^−4^, 10^−3^, 0.01,0.1} for *λ*_1_ and *λ*_2_. The relations between TFR and *λ*_1_, *λ*_2_ on DS1 are shown in Figure 3(d).

After inspecting the trend of TFR with varying *λ*_2_, we can conclude that the term tr⁡(**D**_*c*_^*T*^ **D**_*u*_) in (7) works. In detail, the TFR decreases slowly with the increased *λ*_2_. Once it reaches the minimum point at about 0.01, it begins to ascend rapidly with the increasement of *λ*_2_. Therefore, the suitable range for *λ*_2_ to keep TFR low and stable is about [10^−4^, 10^−2^].

The effect of *λ*_1_ on change detection performance is more apparent than *λ*_2_. Too small or too large *λ*_1_ would degrade the total performance. Even though there are variances of the performance with different *λ*_1_, the TFR is relatively low, and the effective RDD is in the range 0.5 ≤ *λ*_1_ ≤ 2 as can be seen from Figure 3(d). It is worthy noting that if there are abundant computation resources and enough training samples, it is recommended to conduct cross-validation of *λ*_1_ and *λ*_2_ to select the best settings.

#### 4.1.2. Results and Analysis

For visual comparison, the CMs by different methods are shown in Figures 4(e)–4(l), 5(e)–5(l), and 6(e)–6(l). Compared to the other methods, the proposed CD method can obtain better CMs. In detail, pxmsCRF tends to generate over-smoothed CMs, which disables it to accurately capture the edges between the changed and unchanged regions. This phenomenon is particularly remarkable on DS3 (Figure 6(e)). The results of pixel-level CD methods, pxlinSVM and pxrbfSVM, contain lots of salt-and-pepper noise due to the ignorance of the contextual constraints ((f) and (g) of Figures 4–6). The object-level CD methods, splinSVM and sprbfSVM, work much better than the pixel-level counterparts since the local consistency of change is considered. However, as the change features are not discriminative and robust enough, the CMs still have many false and missed alarms ((h) and (i) of Figures 4–6).

For the proposed method, pKNN-CRF, it outperforms all other baseline methods. As can be observed from (l) of Figures 4–6, almost all the changed regions are detected correctly, and the CMs of pKNN-CRF are less noisy. Furthermore, the edges between the changed and unchanged regions nearly align with the true edges.

To conduct the quantitative comparison, the FAR, MAR, and TFR on all data sets are listed in Table 1. From this table, the false alarms and the missed alarms of pKNN-CRF are significantly lower than other related methods, and the improvements can be attributed to the discriminativeness and robustness of the proposed SCD and the robust dictionary-based multiscale region-consistent change decision strategy.

Despite the promising performance of the large-margin classifier in classifying the features, however, as can be seen from both Figures 4–6 and Table 1, the simple prototype KNN classifier combined with the proposed sparse change descriptor is better than SVM equipped with the pixel-level MP features in most cases. This difference demonstrates the effectiveness of the proposed change feature.

As for pKNN and pKNN-CSR, it can be found from Table 1 that the TFRs of pKNN-CSR are lower than the TFRs of pKNN by 1.26%, 1.61%, and 6.10% on DS1, DS2, and DS3, respectively. This indicates that the refined probability maps **r** **P** **M** obtained by the cosparse representation are more accurate than the rough ones **c** **P** **M** estimated by KNN.

When comparing pKNN-CSR and pxmsCRF from Table 1, they have similar TFR on DS1 and DS2. Even so, as mentioned above, pxmsCRF tends to generate excessively smooth CMs. In contrast, by taking the advantage of superpixel-level cosparse representation, pKNN-CSR has the desirable edge-preserving property. Furthermore, by further smoothing the results of pKNN-CSR with CRF, better results (i.e., the results of pKNN-CRF) are obtained.

### 4.2. Second Experiment: Effectiveness Validation of Multiscale Fusion

**Figure 7:**
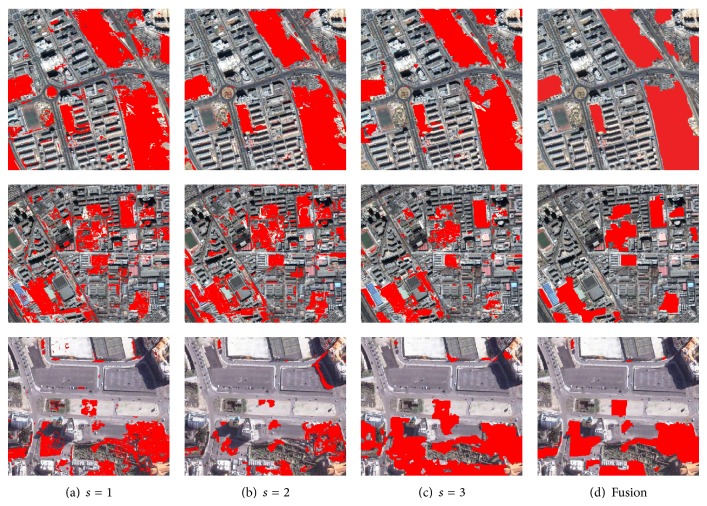
The monoscale and multiscale results on three data sets: DS1, DS2, and DS3 (from up to down).

To validate the effectiveness of multiscale fusion, both the monoscale and the fused results on three data sets are shown in Figure 7. From the figure, with the increased scale, the CMs become smoother. Compared to each monoscale result, the multiscale change detection results have better visual effects. For example, on the multiscale result of DS2, lots of false changes are removed compared to the monoscale results.

The performances of the monoscale and the multiscale results are listed in Table 2. From the table, we can find that the best single-scale result appears at different scales for different data sets. For example, on DS1 the third scale has the lowest TFR, while on DS3 the first scale is optimal. This result implies that it is important to conduct the multiscale analysis for VHR image change detection. By fusing the information from several scales, the change detection performances are greatly enhanced. This is validated by the fact that the fused results obtain the lowest TFR.

### 4.3. Third Experiment: Effectiveness Validation of Feature Extraction

To further demonstrate the effectiveness of the proposed change feature, a comparative experiment is performed on the following change features.

*(i) SCD.* Sparse change descriptor (SCD) is the proposed feature. In this experiment, *m* is set as 7.

*(ii) dfSPEC.* This feature is computed by [**x** ^*T*^, **y** ^*T*^, ‖**x** − **y**‖_2_]^*T*^, where **x** and **y** are the spectral features extracted from the 7 × 7 patches of the bitemporal images **I** and **J**.

*(iii) dfMP.* This feature is extracted with the same manner as dfSPEC, but the spectral feature is replaced by the MP feature with the structuring element parameter *r* = 0,3.

*(iv) dfGB.* This feature is extracted with the same manner as dfSPEC, but the spectral feature is replaced by the Gabor feature with three-scale and six-orientation filters.

*(v) dfUDW.* This feature is extracted with the same manner as dfSPEC, but the feature for each pixel is computed by stacking three-scale undecimated discrete wavelet decomposition coefficients [33].

*(vi) dfSIFT.* This feature is extracted with the same manner as dfSPEC, but the spectral feature is replaced by SIFT [34] feature with a 4 × 4 spatial cell.

All the mentioned features are extracted from the image pyramid of three levels with *ρ* = 2, and they are fed into a classifier for estimating a probability map **P** **M** ^*s*^ for each scale. Summing these maps with the weights computed by (4) generates a finer map **P** **M**, and the final CM is acquired by thresholding **P** **M** with 0.5. Two classifiers are used for probability estimation: the prototype KNN (pKNN) classifier and the nearest neighbor (NN) classifier (preclustering of training samples is not used). In this experiment, the numbers of cluster centers *K*_*c*_ and *K*_*u*_ in pKNN for the changed and unchanged class are set as 500. The number of nearest neighbors for pKNN, *K*, is set by 5-fold cross-validation on the training set.

The performances by different change features are listed in Table 3, from which the proposed change feature SCD is superior to the other change features in most cases. The performance improvement demonstrates the discriminative ability of the proposed change feature, which mainly comes from the sparse-representation-error based change degree description. Specifically, by comparing the performance of SCD and dfMP, the sparse-representation-error based change pattern description is better than the feature-difference-magnitude based one. The underlying reason is that the regression error is more robust to the seasonal variation and registration noise than the Euclidean distance. In short, the combination of the change degree description and the change pattern description is important for improving the separability of change feature.

### 4.4. Fourth Experiment: Effectiveness Validation of RDDL

To demonstrate the effectiveness of RDD, three other kinds of dictionaries are used for comparison.

*(i) OrigD.* This dictionary is learned by the model in (7) on the original training sets with the weights of reconstruction term that are set as 1 and *λ*_2_ = 0.

*(ii) AugD.* This dictionary is learned by the model in (7) on the augmented training sets with the weights of reconstruction term that are set as 1 and *λ*_2_ = 0.

*(iii) AugDw.* This dictionary is learned by the model in (7) on the augmented training sets with *λ*_2_ = 0. In this case, the weights are set by the method in Section 2.3.2 and their values are not updated.

*(iv) RDD.* This is the proposed robust discriminative dictionary, which is learned by the model in (7) on the augmented training sets. Note that the weights are allowed to be updated.

Obviously, OrigD is learned on the original training data. AugD and AugDw are learned on the augmented training data without and with weighting, respectively.

To evaluate the performances of these dictionaries quantitatively, FAR, MAR, and TFR are computed for the CMs generated by pKNN-CSR. The parameters of pKNN are set as *K*_*c*_ = *K*_*u*_ = 500 and *K* = 7. For fair comparison, *λ*_1_ and *λ*_2_ are tuned to obtain the lowest TFRs for each type of dictionary and the best performances are reported. The searching range for *λ*_1_ is [10^−3^, 2] and that for *λ*_2_ is [10^−4^, 1].

The results of different dictionaries are shown in Table 4. From Table 4, the proposed dictionary, RDD, performs best in terms of TFR. For DS1, the labeled data are representative enough to predict the probabilities of the unlabeled data belonging to the changed class with a relatively high accuracy. However, there are still some outliers that may degrade the discriminative ability of the dictionaries. Thus, the TFR of AugD is higher than OrigD. Because the correct probability prediction weakens the impact from the outliers by weighting, the TFR of AugDw matches that of OrigD and is lower than that of AugD. For RDD, the updated weights enable it to recognize more outliers than AugDw and the discriminativeness of the learned dictionaries is improved.

On DS2, the labeled samples are too scarce to acquire a precise probability map, which leads to the high TFR of OrigD. The outliers contained in the extra samples prevent AugD to acquire lower TFR than OrigD. For AugDw, as some samples are recognized as outliers and they are assigned with small weights, the TFR is much lower than that of OrigD and AugD. For the robust learning manner in RDD, more outliers are removed by gradually reducing the weights. This makes RDD achieve the lowest TFR. Similar analysis can be conducted on DS3.

In summary, the initially unlabeled data helps improve the abundance of training data and thus reduces the missed changes. Moreover, the robustness of the RDDL model helps to remove the mislabeled data and keep the FAR low.

## 5. Conclusions

This paper proposes a supervised change detection approach for VHR images. It includes two parts: sparse change descriptor extraction and multiscale region-level change decision. The sparse change descriptor integrates the change degree and change pattern description, which improves the discriminative ability of the change feature; meanwhile it makes it robust to false changes. On the other hand, the multiscale region-level change decision strategy enables the proposed change detection method to detect the changes from different scales and reduce the salt-and-pepper noise of change maps. Experiments on several data sets demonstrate the superiority of the proposed change feature and the change decision procedure.

## Figures and Tables

**Table 1:** Performance comparison of different CD methods. The bold value is the best and the italic one is the second.

Data set	Method	FAR (%)	MAR (%)	TFR (%)	Method	FAR (%)	MAR (%)	TFR (%)
DS1	pxmsCRF	4.04	12.96	*6.60 *	pxlinSVM	19.24	85.32	38.20
	pxrbfSVM	6.39	18.33	9.81	splinSVM	9.72	26.59	14.56
	sprbfSVM	4.76	16.91	8.24	pKNN	2.10	22.99	8.09
	pKNN-CSR	2.54	17.50	*6.83 *	pKNN-CRF	0.61	18.56	**5.76**

DS2	pxmsCRF	7.13	18.85	*8.89 *	pxlinSVM	9.42	41.23	14.19
	pxrbfSVM	14.18	19.49	14.97	splinSVM	3.32	48.51	10.11
	sprbfSVM	5.67	31.14	9.49	pKNN	6.87	29.63	10.29
	pKNN-CSR	5.74	25.34	*8.68 *	pKNN-CRF	1.84	24.48	**5.24**

DS3	pxmsCRF	20.76	7.20	18.95	pxlinSVM	9.96	65.42	17.36
	pxrbfSVM	12.07	34.73	15.09	splinSVM	3.63	59.77	11.13
	sprbfSVM	0.40	83.81	11.53	pKNN	13.93	11.85	13.65
	pKNN-CSR	5.77	19.04	*7.55 *	pKNN-CRF	3.25	11.03	**4.29**

**Table 2:** Performance evaluation of the monoscale and the multiscale results. The bold value is the best and the italic one is the second.

Data set	**r** **P** **M** ^1^ > 0.5			**r** **P** **M** ^2^ > 0.5			**r** **P** **M** ^3^ > 0.5			Fusion		
	FAR (%)	MAR (%)	TFR (%)	FAR (%)	MAR (%)	TFR (%)	FAR (%)	MAR (%)	TFR (%)	FAR (%)	MAR (%)	TFR (%)
DS1	7.28	21.53	11.37	2.62	23.45	8.60	3.39	19.02	*7.87 *	0.61	18.56	**5.76**
DS2	9.69	31.48	12.96	10.22	28.26	12.93	4.89	39.09	*10.02 *	1.84	24.48	**5.24**
DS3	4.87	27.75	*7.92 *	2.49	53.29	9.28	14.46	17.62	14.89	3.25	11.03	**4.29**

**Table 3:** Performance comparison of different change features.

Data set	Feature	pKNN			NN		
		FAR (%)	MAR (%)	TFR (%)	FAR (%)	MAR (%)	TFR (%)
DS1	SCD	2.40	19.39	**7.27**	3.22	17.42	**7.30**
	dfSPEC	7.96	24.81	12.79	10.52	21.49	13.67
	dfMP	6.82	27.89	12.87	6.05	23.14	*10.95 *
	dfGB	5.52	27.73	*11.89 *	5.86	21.90	*10.46 *
	dfUDW	8.95	28.24	14.48	7.96	24.16	12.61
	dfSIFT	4.36	30.60	*11.88 *	6.47	26.13	12.11

DS2	SCD	7.51	30.39	*10.94 *	8.89	22.74	**10.97**
	dfSPEC	9.15	29.93	12.27	7.81	28.77	**10.96**
	dfMP	10.75	29.48	13.56	7.84	25.15	**10.44**
	dfGB	10.29	31.33	13.45	8.00	28.41	11.07
	dfUDW	13.11	39.33	17.05	9.55	35.98	13.52
	dfSIFT	3.00	44.22	**9.19**	6.42	35.59	**10.80**

DS3	SCD	12.10	13.52	**12.29**	10.51	16.42	**11.30**
	dfSPEC	13.02	21.20	14.12	11.83	20.45	12.98
	dfMP	12.04	29.66	14.39	8.81	34.33	*12.22 *
	dfGB	14.24	26.02	15.81	11.82	28.01	13.98
	dfUDW	17.43	28.36	18.89	14.15	31.10	16.42
	dfSIFT	8.87	41.30	*13.20 *	10.63	35.65	13.97

**Table 4:** Performance comparison of different dictionaries.

Dataset	Dictionary	FAR (%)	MAR (%)	TFR (%)
DS1	OrigD	2.28	18.13	*6.82 *
	AugD	3.62	16.35	7.28
	AugDw	2.36	17.86	*6.81 *
	RDD	2.36	16.57	**6.43**

DS2	OrigD	5.69	36.4	10.30
	AugD	7.70	30.04	11.05
	AugDw	6.01	29.53	*9.54 *
	RDD	5.74	25.34	**8.68**

DS3	OrigD	6.98	27.21	9.68
	AugD	4.52	49.02	10.46
	AugDw	5.40	29.67	*8.64 *
	RDD	5.07	21.55	**7.27**
